# Supplementary material for: Two-Step One-Pot Reductive Amination of Furanic Aldehydes Using CuAlOx Catalyst in a Flow Reactor
Source: Molecules. 2020 Oct 17;25(20):4771. doi: 10.3390/molecules25204771 (PMC7594031; doi:10.3390/molecules25204771)
Supplement: Supplementary file 1 [file molecules-25-04771-s001.pdf]

Supplementary Materials

Two-step one-pot reductive amination of furanic aldehydes using $CuAlO_x$ catalyst in a flow reactor

Alexey L. Nuzhdin,* Marina V. Bukhtiyarova and Valerii I. Bukhtiyarov

Boreskov Institute of Catalysis SB RAS, Lavrentieva Ave. 5, 630090 Novosibirsk (Russia)

E-mail: anuzhdin@catalysis.ru

1. Condensation of furanic aldehydes with aniline

2. Catalytic stability

3. NMR data

1. Condensation of furanic aldehydes with aniline

The condensation of HMF (or AMF) with aniline was investigated by gas chromatography (GC) on Agilent 6890N instrument equipped with a JAS 2370AA atomic emission detector (AED) and a capillary HP-1MS column (30 m × 0.32 mm × 1.00 μm). The emission line for carbon atoms at 179 nm was used [I.V. Deliy, E.N. Vlasova, A.L. Nuzhdin, E.Yu. Gerasimov, G.A. Bukhtiyarova, RSC Adv., 2014, 4, 2242].

It should be noted that the study of imine hydrogenation products by gas chromatography is impossible due to the decomposition of aminomethylhydroxymethylfuran derivatives (compounds **2** and **6**) during the evaporation of the sample.

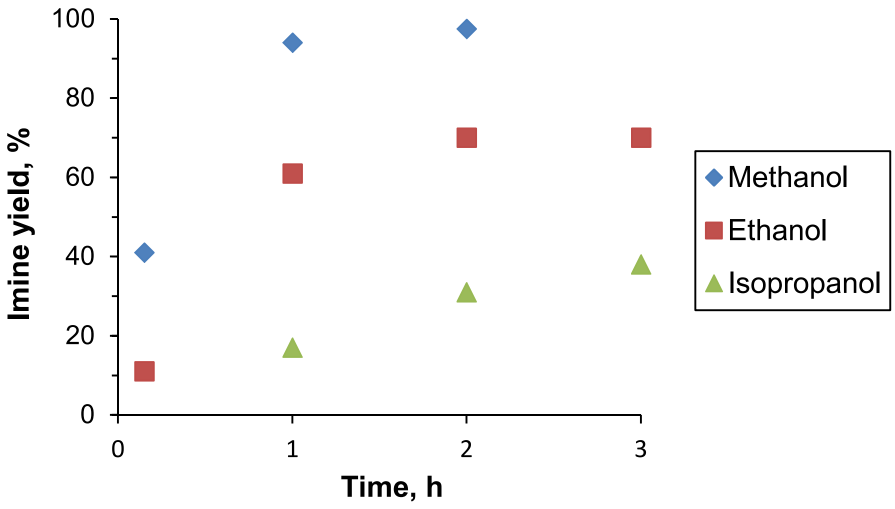

Figure S1. The time dependence of imine yield in the condensation of HMF (0.05 M) and aniline (0.05 M) at room temperature

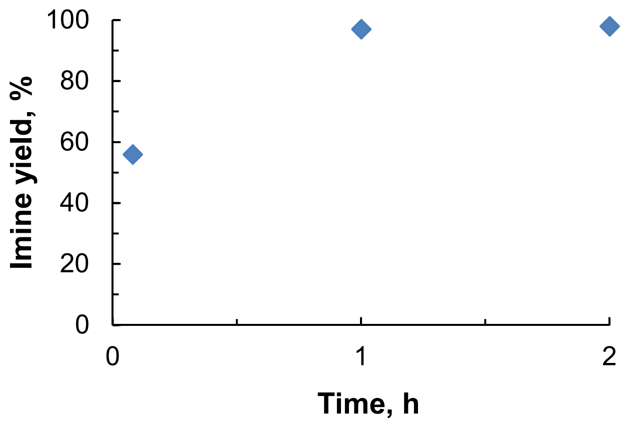

Figure S2. The time dependence of imine yield in the reaction between AMF (0.05 M) and aniline (0.05 M) in methanol at room temperature

2. Catalytic stability

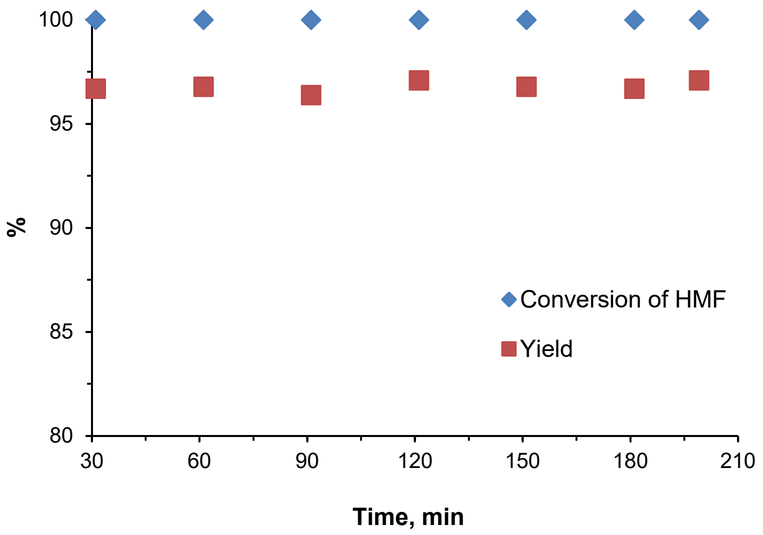

Figure S3. Time dependence of **2a** yield in the two-step one-pot reductive amination of HMF (0.05M) with aniline (0.05M) at 100 °C

3. NMR data

^{1}H NMR spectra were recorded at 500.03 MHz on a Bruker Avance III 500 spectrometer using chloroform-*d* (99.8 atom % D, Aldrich) as a solvent. The chemical shifts are reported in ppm of the δ scale and referred to signal of the solvent (δ = 7.26 ppm for residual protons). ^{1}H assignment abbreviations are the following: singlet (s), doublet (d), triplet (t), multiplet (m) and broad singlet (brs).

^{1}H NMR spectrum of the final reaction mixture for **Table 2 Entry 1**:

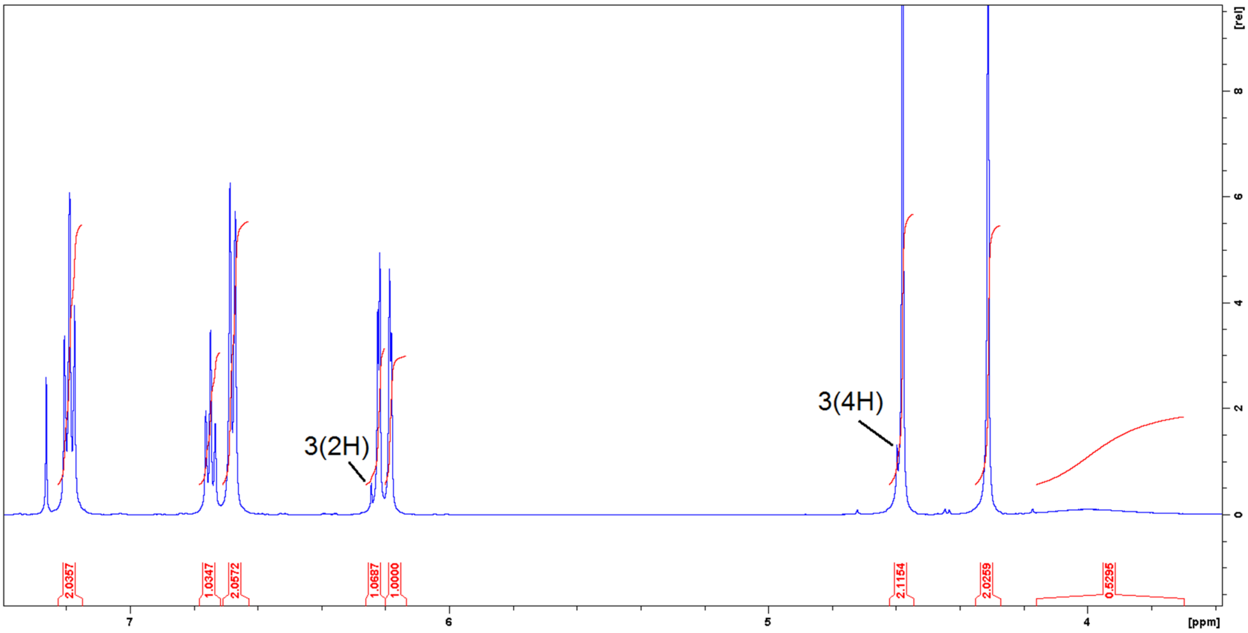

The spectrum contains peaks of compounds: **2a** (main product) and **3**.

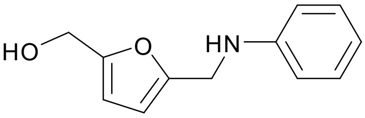

(2a)

^{1}H NMR (500 MHz, $CDCl_3$) δ ppm: 4.00 (brs, 1H) 4.31 (s, 2H), 4.58 (s, 2H), 6.18 (d, J=3.1 Hz, 1H), 6.22 (d, J=3.1 Hz, 1H), 6.68 (d, J=7.9 Hz, 2H), 6.75 (t, J=7.3 Hz, 1H), 7.16-7.22 (m, 2H).

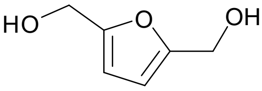

(3)

^{1}H NMR (500 MHz, $CDCl_3$) δ ppm: 4.59 (s, 4H), 6.24 (s, 2H).

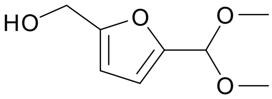

(4)

^{1}H NMR (500 MHz, $CDCl_3$) δ ppm: 3.37 (s, 6H), 4.59 (s, 2H), 5.40 (s, 1H), 6.27 (d, J=3.0 Hz, 1H), 6.37 (d, J=3.0 Hz, 1H).

^{1}H NMR spectrum of the final reaction mixture for **Table 2 Entry 5**:

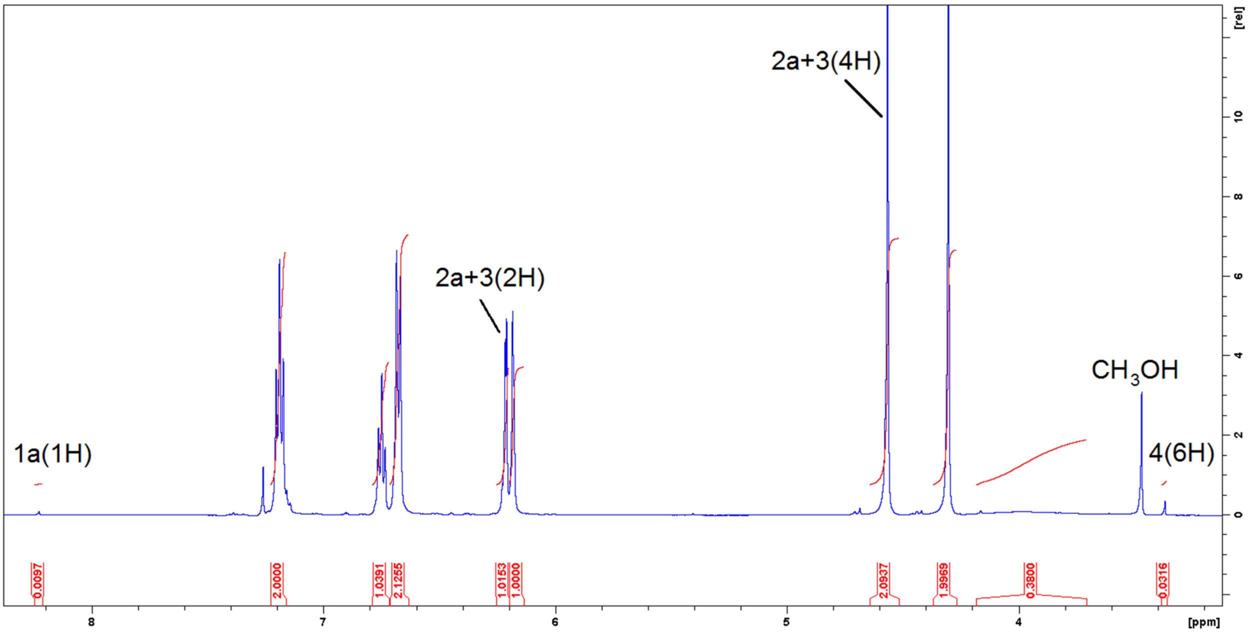

The spectrum contains peaks of compounds: **2a** (main product), **1a**, **3** and **4**.

^{1}H NMR spectrum of the final reaction mixture for **Table 2 Entry 6**:

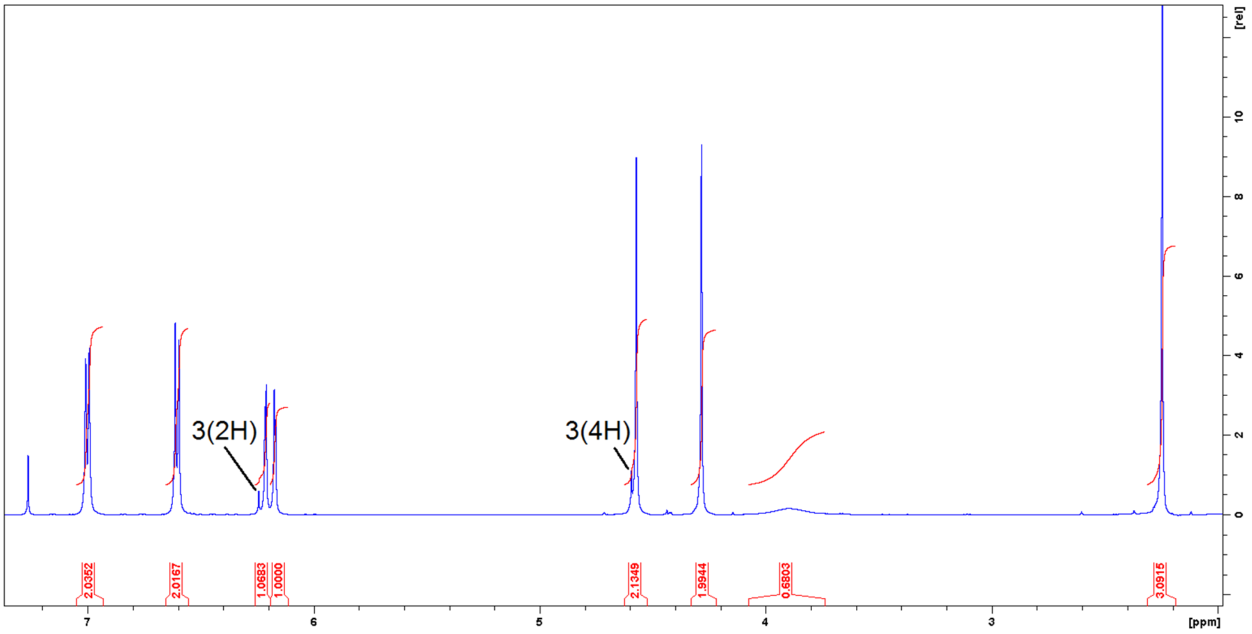

The spectrum contains peaks of compounds: **2b** (main product) and **3**.

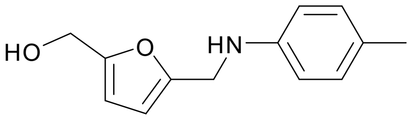

(2b)

^{1}H NMR (500 MHz, $CDCl_3$) δ ppm: 2.24 (s, 3H), 3.90 (brs, 1H), 4.28 (s, 2H), 4.57 (s, 2H), 6.17 (d, *J*=3.0 Hz, 1H), 6.21 (d, *J*=3.0 Hz, 1H), 6.60 (d, *J*=8.3 Hz, 2H), 7.00 (d, *J*=8.2 Hz, 2H).

^{1}H NMR spectrum of the final reaction mixture for **Table 2 Entry 7**:

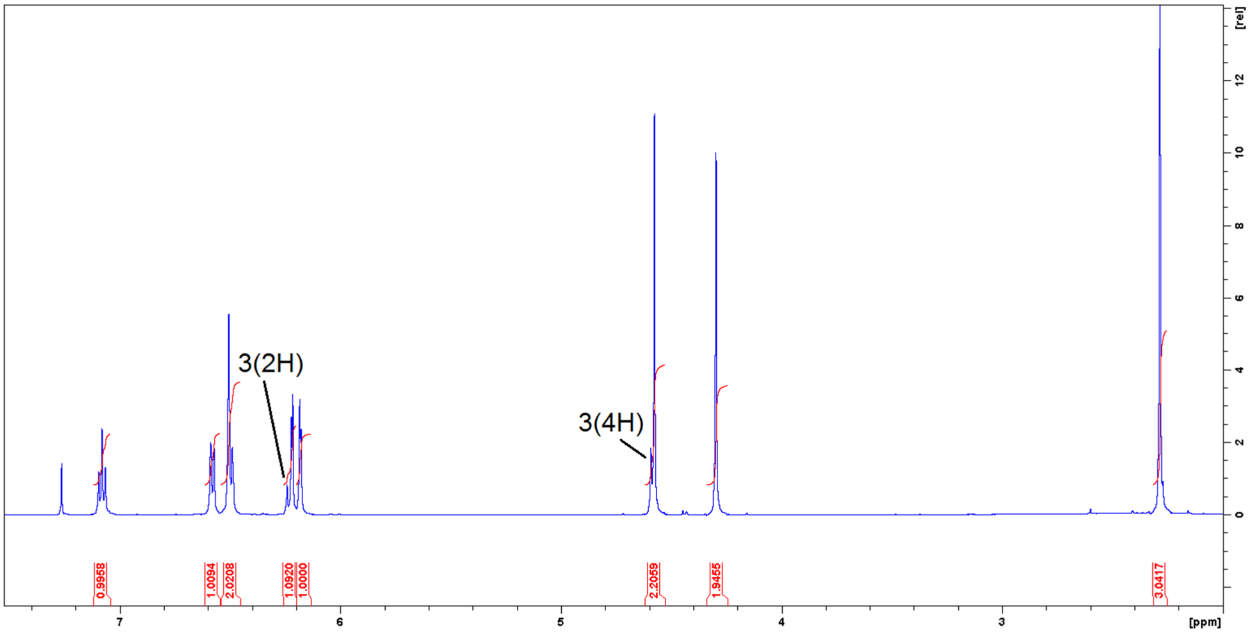

The spectrum contains peaks of compounds: **2c** (main product) and **3**.

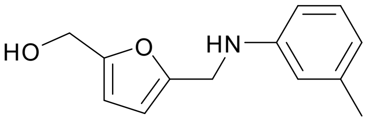

(2c)

^{1}H NMR (500 MHz, $CDCl_3$) δ ppm: 2.28 (s, 3H), 4.30 (s, 2H), 4.58 (s, 2H), 6.18 (d, *J*=3.0 Hz, 1H), 6.22 (d, *J*=3.0 Hz, 1H), 6.47-6.52 (m, 2H), 6.58 (d, *J*=7.5 Hz, 1H), 7.08 (t, *J*=7.6 Hz, 1H).

^{1}H NMR spectrum of the final reaction mixture for **Table 2 Entry 8**:

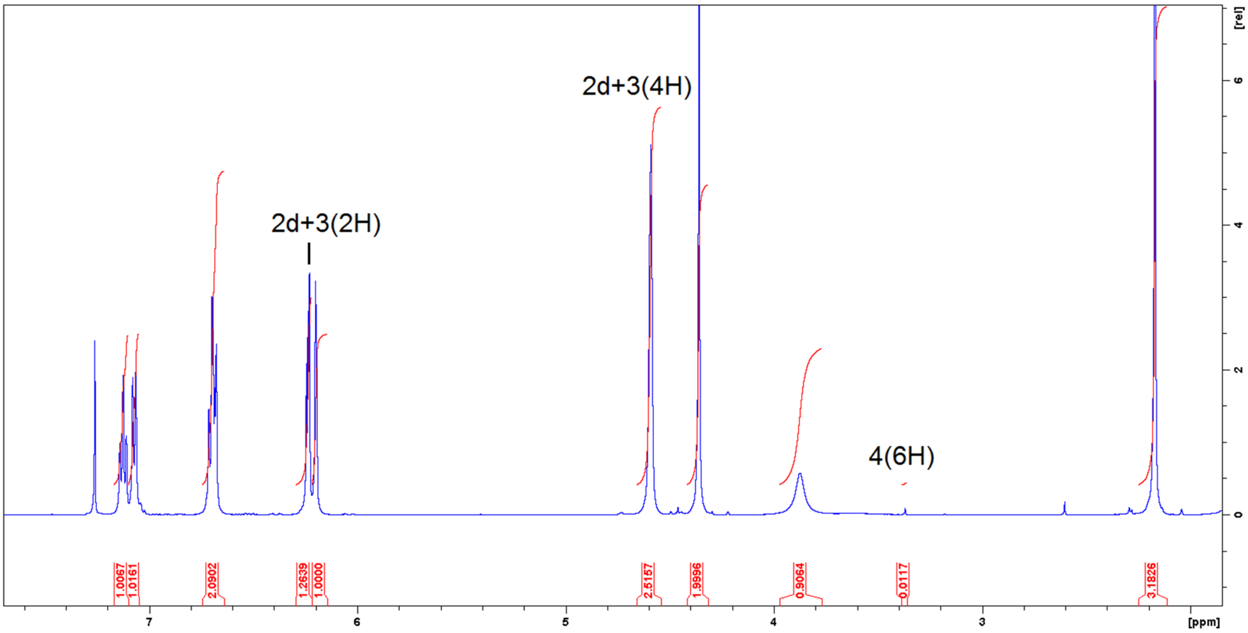

The spectrum contains peaks of compounds: **2d** (main product), **3** and **4**.

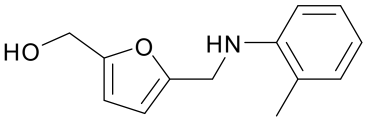

(2d)

^{1}H NMR (500 MHz, $CDCl_3$) δ ppm: 2.17 (s, 3H), 3.88 (brs, 1H), 4.36 (s, 2H), 4.59 (s, 2H), 6.20 (d, *J*=3.0 Hz, 1H), 6.23 (d, *J*=3.0 Hz, 1H), 6.65-6.74 (m, 2H), 7.07 (d, *J*=7.2 Hz, 1H), 7.13 (t, *J*=7.6 Hz, 1H).

^{1}H NMR spectrum of the final reaction mixture for **Table 2 Entry 9**:

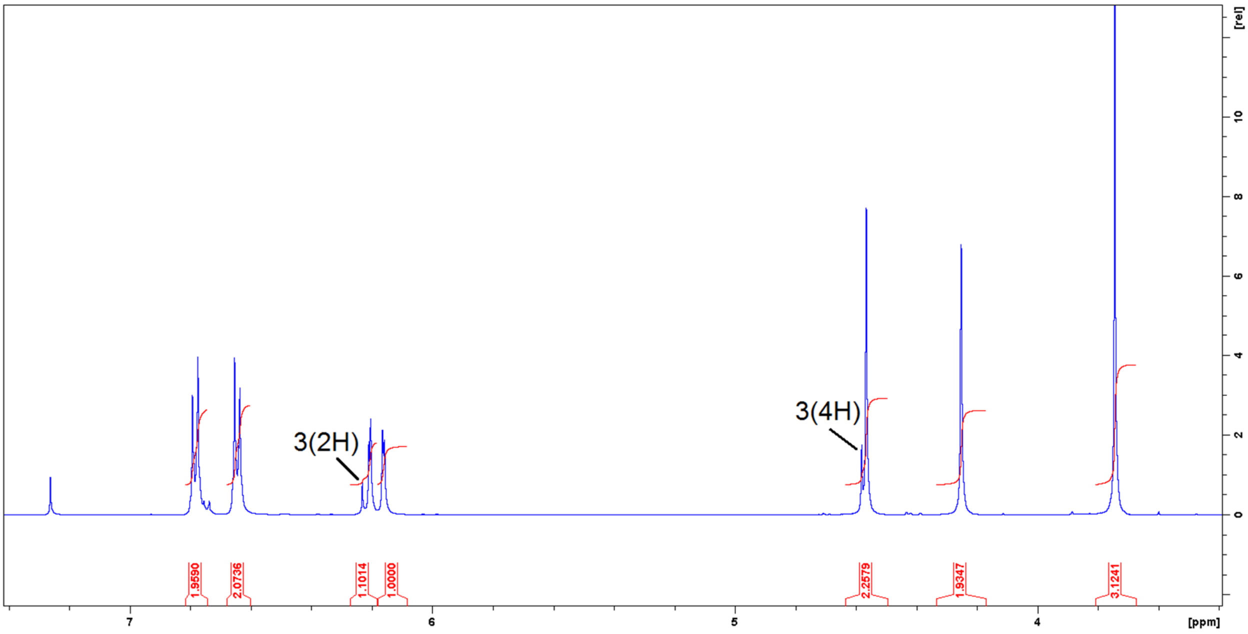

The spectrum contains peaks of compounds: **2e** (main product), **3** and *p*-anisidine.

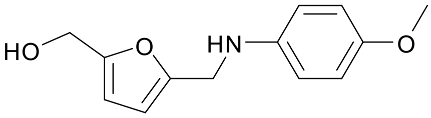

(2e)

^{1}H NMR (500 MHz, $CDCl_3$) δ ppm: 3.75 (s, 3H), 4.25 (s, 2H), 4.57 (s, 2H), 6.16 (d, *J*=2.9 Hz, 1H), 6.21 (d, *J*=2.9 Hz, 1H), 6.65 (d, *J*=8.8 Hz, 2H), 6.78 (d, *J*=8.8 Hz, 2H).

^{1}H NMR spectrum of the final reaction mixture for **Table 2 Entry 10**:

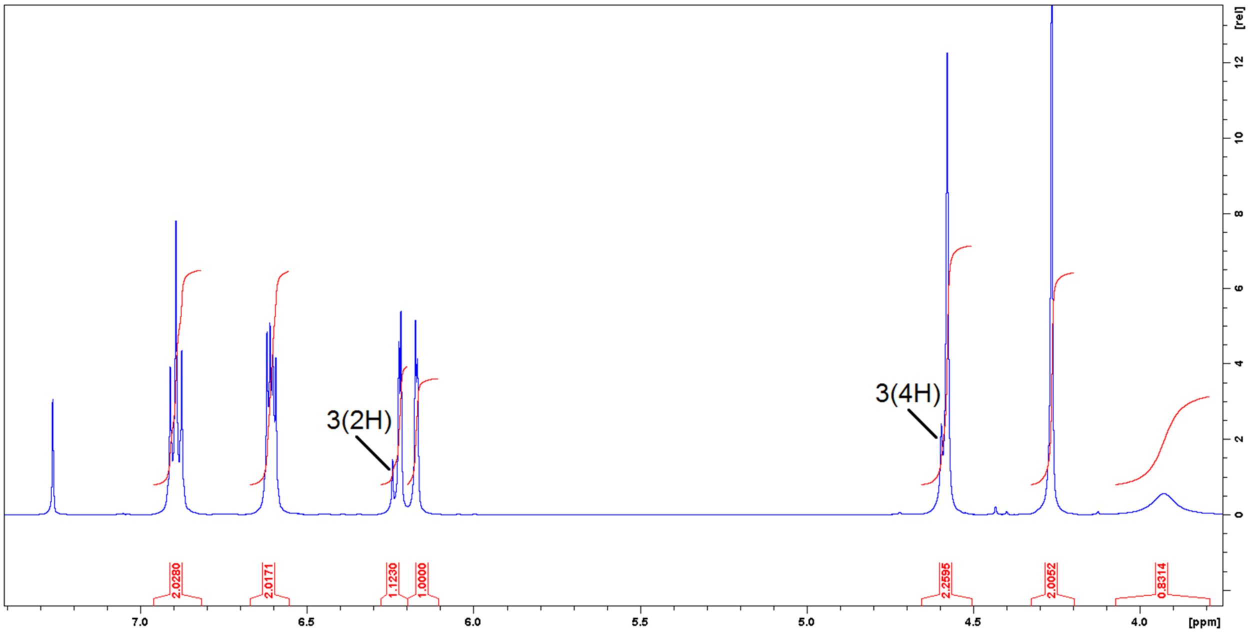

The spectrum contains peaks of compounds: **2f** (main product) and **3**.

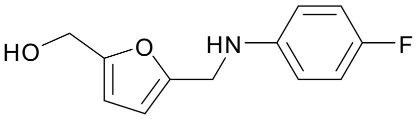

(2f)

^{1}H NMR (500 MHz, $CDCl_3$) δ ppm: 3.93 (brs, 1H), 4.27 (s, 2H), 4.58 (s, 2H), 6.17 (d, *J*=3.0 Hz, 1H), 6.22 (d, *J*=3.0 Hz, 1H), 6.58-6.63 (m, 2H), 6.89 (t, *J*=8.6 Hz, 2H).

^{1}H NMR spectrum of the final reaction mixture for **Table 2 Entry 11**:

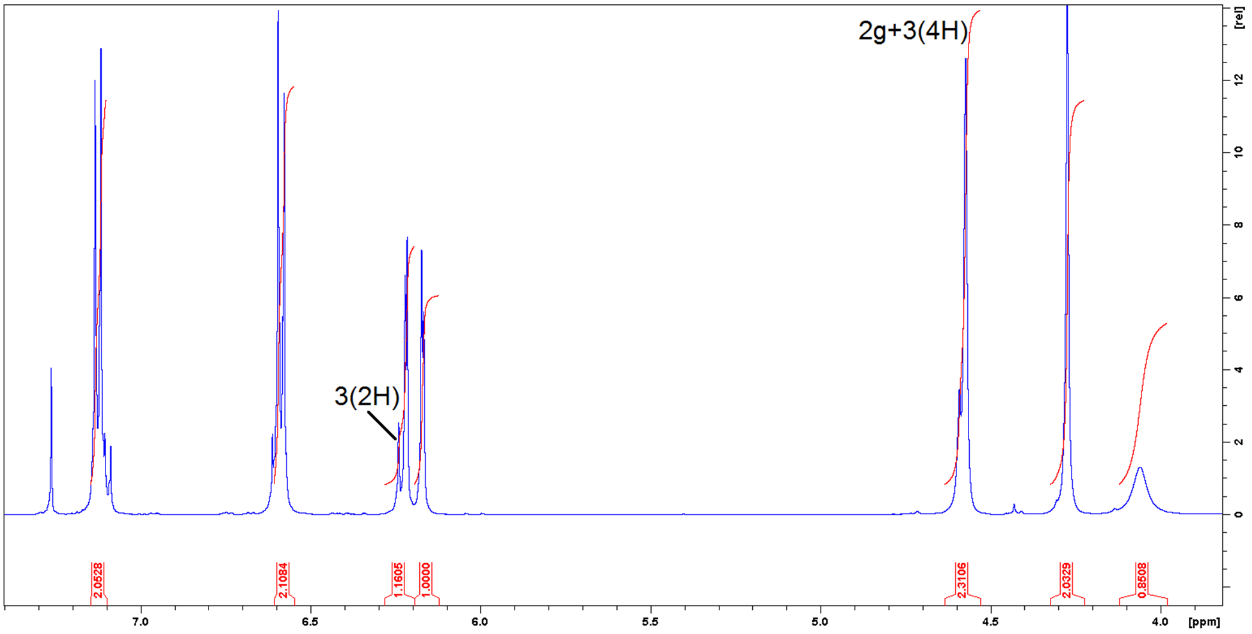

The spectrum contains peaks of compounds: **2g** (main product), **3** and *p*-chloroaniline.

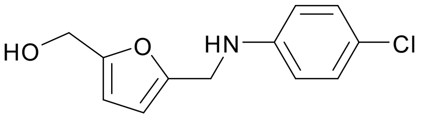

(2g)

^{1}H NMR (500 MHz, $CDCl_3$) δ ppm: 4.06 (brs, 1H), 4.27 (s, 2H), 4.57 (s, 2H), 6.17 (d, *J*=3.0 Hz, 1H), 6.22 (d, *J*=3.0 Hz, 1H), 6.59 (d, *J*=8.8 Hz, 2H), 7.12 (d, *J*=8.8 Hz, 2H).

^{1}H NMR spectrum of the final reaction mixture for **Table 2 Entry 12**:

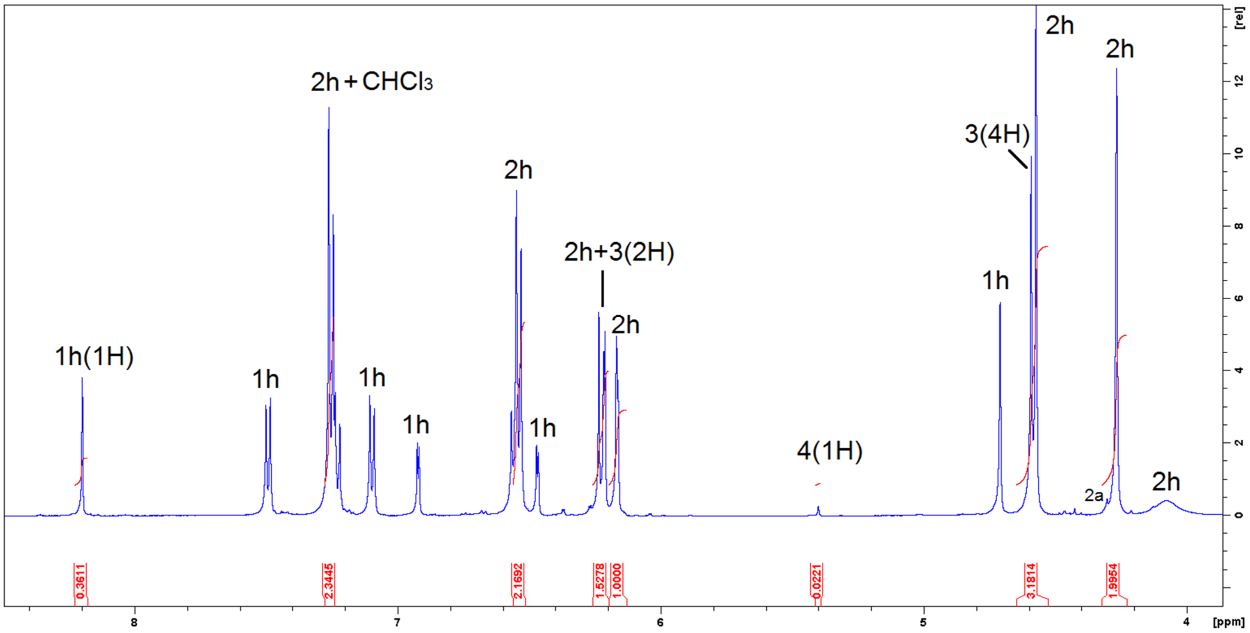

The spectrum contains peaks of compounds: **2h**, **1h**, **3**, **4**, *p*-bromoaniline and **2a**.

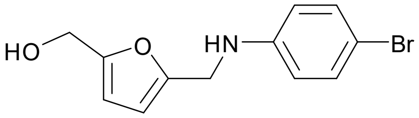

(2h)

^{1}H NMR (500 MHz, $CDCl_3$) δ ppm: 4.08 (brs, 1H), 4.27 (s, 2H), 4.57 (s, 2H), 6.17 (d, *J*=3.0 Hz, 1H), 6.22 (d, *J*=3.0 Hz, 1H), 6.54 (d, *J*=8.8 Hz, 2H), 7.25 (d, *J*=8.8 Hz, 2H).

^{1}H NMR spectrum of the final reaction mixture for **Table 2 Entry 14**:

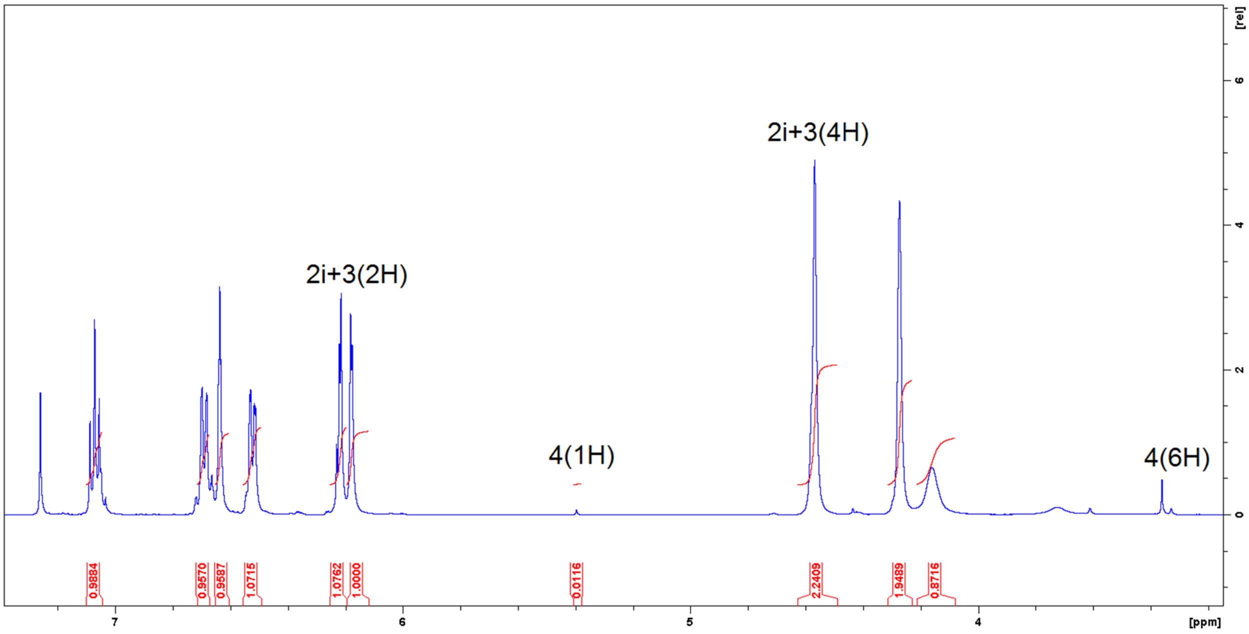

The spectrum contains peaks of compounds: **2i** (main product), **3, 4** and *m*-chloroaniline.

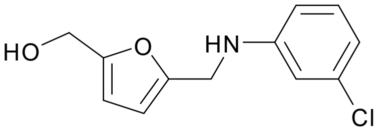

(2i)

^{1}H NMR (500 MHz, $CDCl_3$) δ ppm: 4.16 (brs, 1H), 4.27 (s, 2H), 4.57 (s, 2H), 6.18 (d, *J*=3.0 Hz, 1H), 6.22 (d, *J*=3.0 Hz, 1H), 6.50-6.55 (m, 1H), 6.64 (t, *J*=1.9 Hz, 1H), 6.67-6.71 (m, 1H), 7.07 (t, *J*=8.1 Hz, 1H).

^{1}H NMR spectrum of the final reaction mixture for **Table 2 Entry 15**:

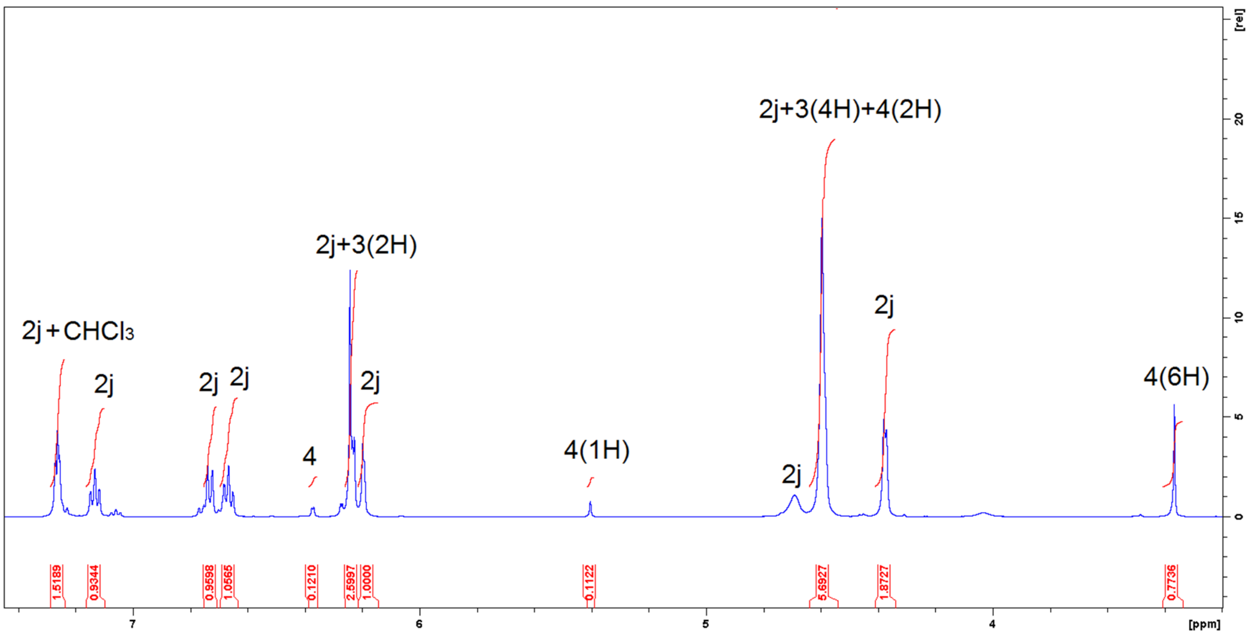

The spectrum contains peaks of compounds: **2j**, **3** and **4** and *o*-chloroaniline.

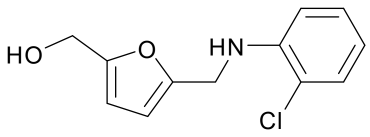

(2j)

^{1}H NMR (500 MHz, $CDCl_3$) δ ppm: 4.37 (d, *J*=4.4 Hz, 2H), 4.60 (s, 2H), 4.69 (brs, 1H), 6.19 (d, *J*=3.0 Hz, 1H), 6.23 (d, *J*=3.0 Hz, 1H), 6.63-6.70 (m, 1H), 6.71-6.76 (m, 1H), 7.10-7.16 (m, 1H), 7.24-7.29 (m, 1H).

^{1}H NMR spectrum of the final reaction mixture for **Table 2 Entry 16**:

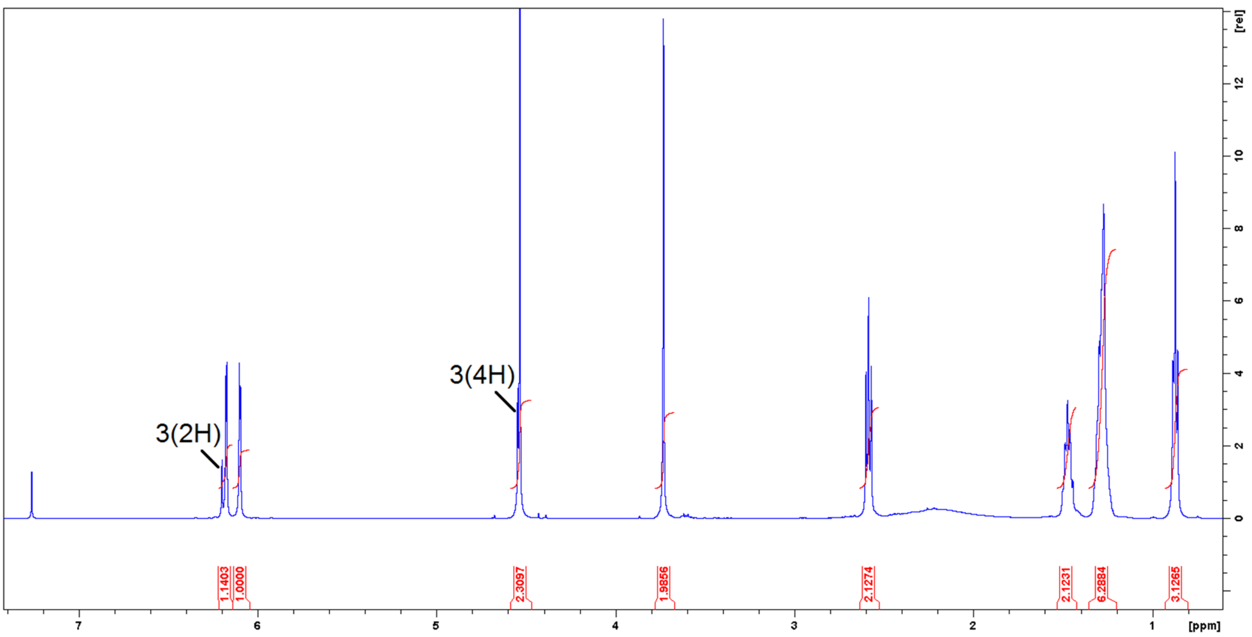

The spectrum contains peaks of compounds: **2k** (main product) and **3**.

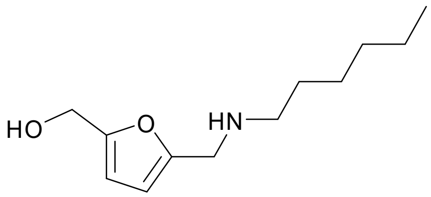

(2k)

^{1}H NMR (500 MHz, $CDCl_3$) δ ppm: 0.84-0.91 (m, 3H), 1.21-1.34 (m, 6H), 1.43-1.51 (m, 2H), 2.59 (t, *J*=7.3 Hz, 2H), 3.73 (s, 2H), 4.53 (s, 2H), 6.10 (d, *J*=3.0 Hz, 1H), 6.18 (d, *J*=3.0 Hz, 1H).

^{1}H NMR spectrum of the final reaction mixture for **Table 3 Entry 1**:

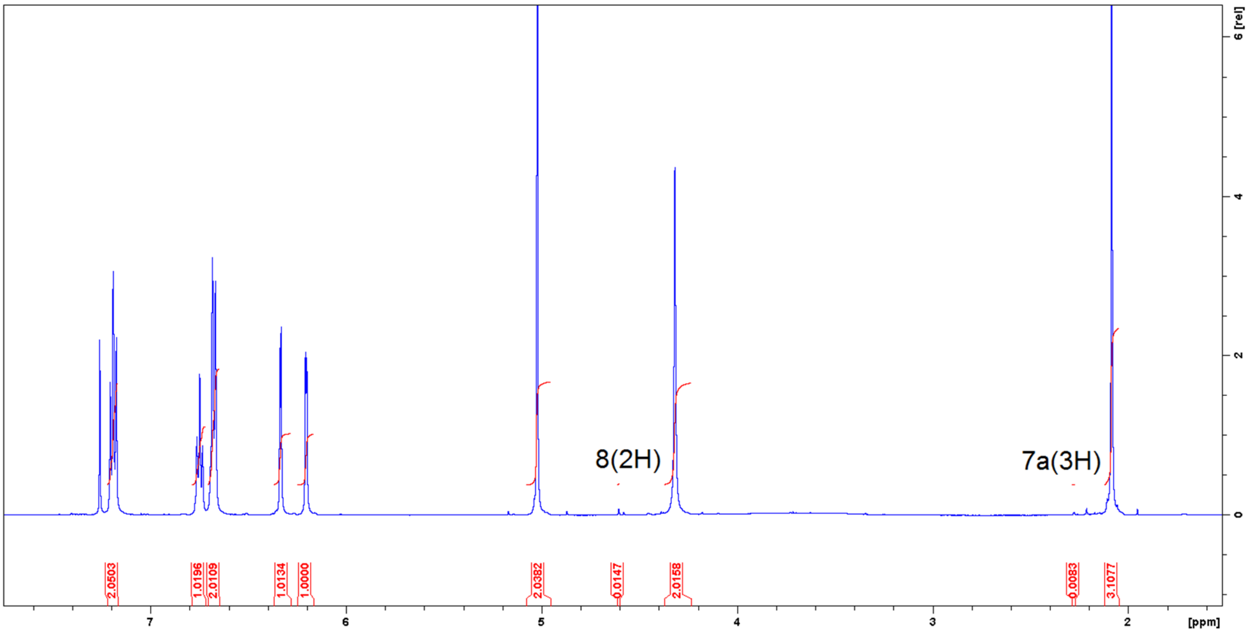

The spectrum contains peaks of compounds: **6a** (main product), **8** and **7a**.

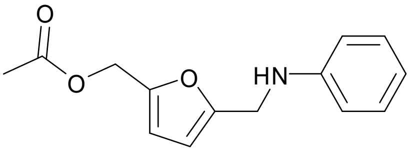

(6a)

^{1}H NMR (500 MHz, $CDCl_3$) δ ppm: 2.09 (s, 3H), 4.32 (s, 2H), 5.02 (s, 2H), 6.21 (d, *J*=3.0 Hz, 1H), 6.34 (d, *J*=3.0 Hz, 1H), 6.68 (d, *J*=7.8 Hz, 2H), 6.75 (t, *J*=7.4 Hz, 1H), 7.16-7.22 (m, 2H).

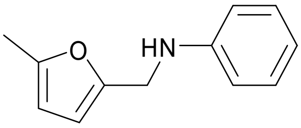

(7a)

^{1}H NMR (500 MHz, $CDCl_3$) δ ppm: 2.28 (s, 3H), 4.26 (s, 2H), 5.90 (d, *J*=2.8 Hz, 1H), 6.11 (d, *J*=2.8 Hz, 1H), 6.68 (d, *J*=7.7 Hz, 2H), 6.74 (t, *J*=7.3 Hz, 1H), 7.16-7.22 (m, 2H).

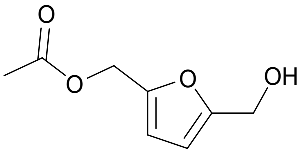

(8)

^{1}H NMR (500 MHz, $CDCl_3$) δ ppm: 2.08 (s, 3H), 4.60 (s, 2H), 5.03 (s, 2H), 6.26 (d, *J*=3.0 Hz, 1H), 6.36 (d, *J*=3.0 Hz, 1H).

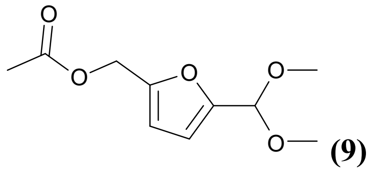

(9)

^{1}H NMR (500 MHz, $CDCl_3$) δ ppm: 2.07 (s, 3H), 3.37 (s, 6H), 5.03 (s, 2H), 5.41 (s, 1H), 6.37-6.40 (m, 2H).

^{1}H NMR spectrum of the final reaction mixture for **Table 3 Entry 2**:

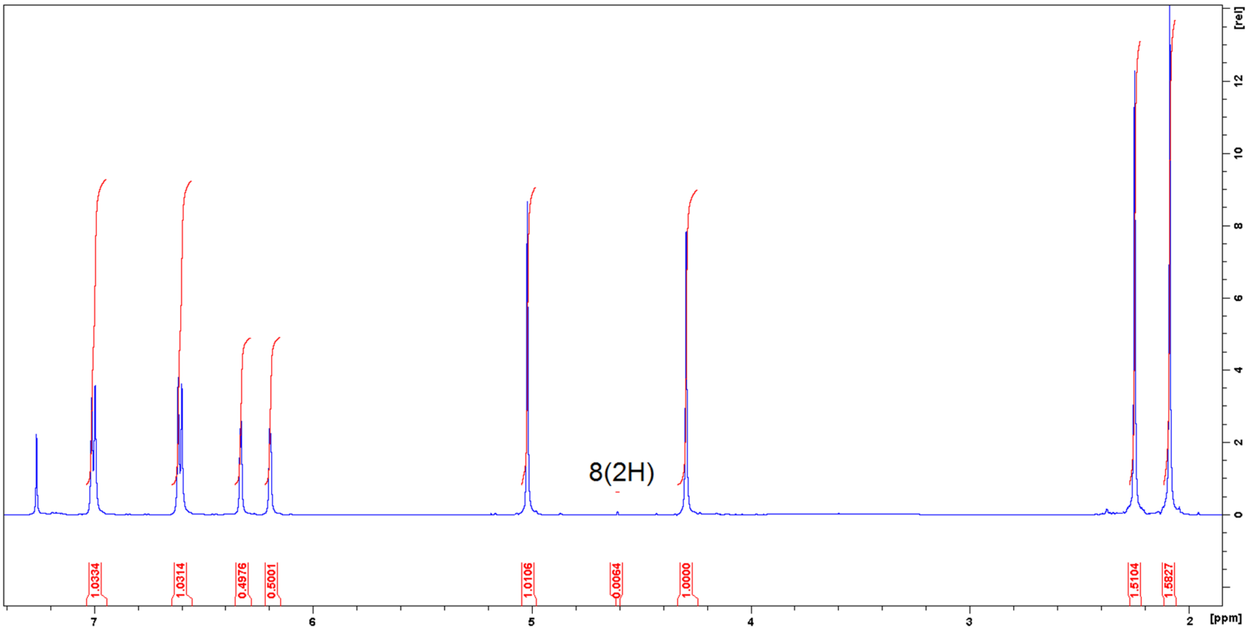

The spectrum contains peaks of compounds: **6b** (main product) and **8**.

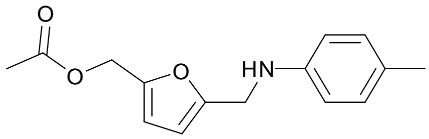

(6b)

^{1}H NMR (500 MHz, $CDCl_3$) δ ppm: 2.09 (s, 3H), 2.25 (s, 3H), 4.29 (s, 2H), 5.02 (s, 2H), 6.19 (d, *J*=3.0 Hz, 1H), 6.33 (d, *J*=3.0 Hz, 1H), 6.61 (d, *J*=8.3 Hz, 2H), 7.00 (d, *J*=8.1 Hz, 2H).

^{1}H NMR spectrum of the final reaction mixture for **Table 3 Entry 3**:

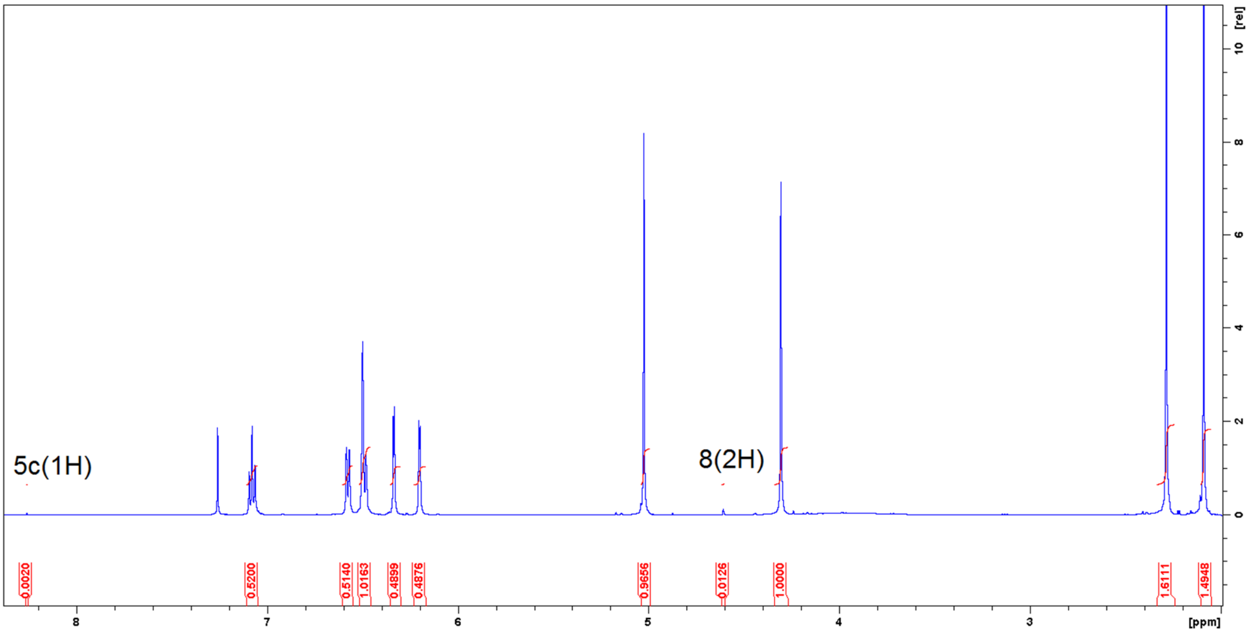

The spectrum contains peaks of compounds: **6c** (main product), **5c** and **8**.

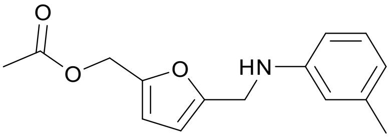

(6c)

^{1}H NMR (500 MHz, $CDCl_3$) δ ppm: 2.09 (s, 3H), 2.28 (s, 3H), 4.31 (s, 2H), 5.02 (s, 2H), 6.20 (d, *J*=3.0 Hz, 1H), 6.34 (d, *J*=3.0 Hz, 1H), 6.47-6.52 (m, 2H), 6.58 (d, *J*=7.6 Hz, 1H), 7.08 (t, *J*=7.6 Hz, 1H).

^{1}H NMR spectrum of the final reaction mixture for **Table 3 Entry 4**:

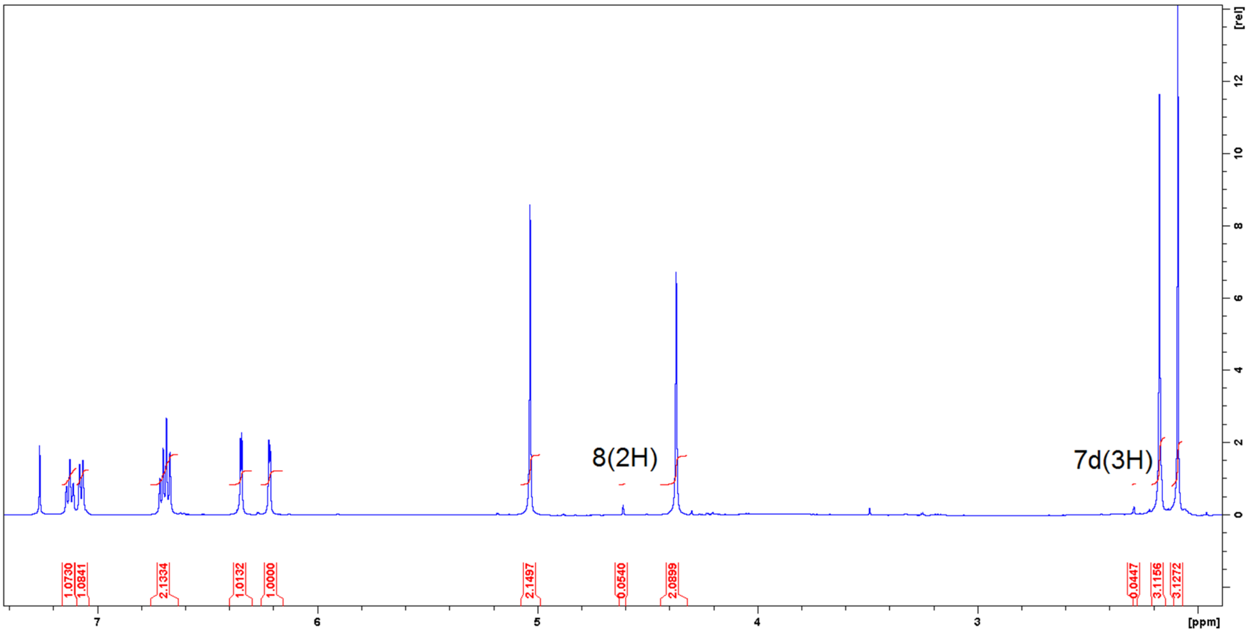

The spectrum contains peaks of compounds: **6d** (main product), **7d** and **8**.

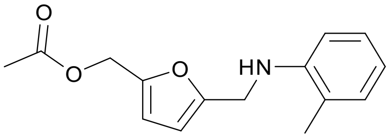

(6d)

^{1}H NMR (500 MHz, $CDCl_3$) δ ppm: 2.09 (s, 3H), 2.17 (s, 3H), 4.37 (s, 2H), 5.03 (s, 2H), 6.22 (d, *J*=3.0 Hz, 1H), 6.35 (d, *J*=3.0 Hz, 1H), 6.65-6.72 (m, 2H), 7.07 (d, *J*=7.4 Hz, 1H), 7.12 (t, *J*=7.6 Hz, 1H).

^{1}H NMR spectrum of the final reaction mixture for **Table 3 Entry 5**:

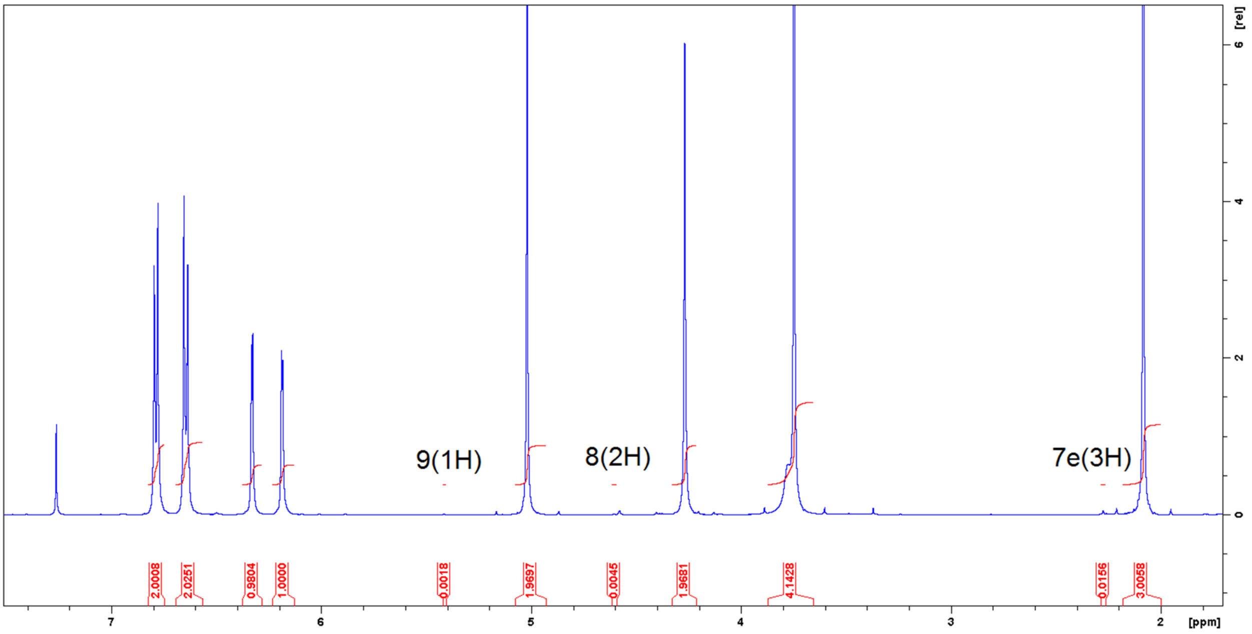

The spectrum contains peaks of compounds: **6e** (main product), **7e, 8** and **9**.

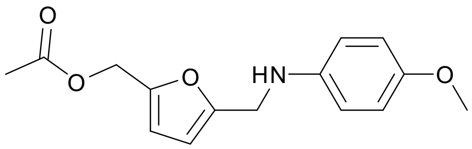

(6e)

^{1}H NMR (500 MHz, $CDCl_3$) δ ppm: 2.08 (s, 3H), 3.75 (s, 3H), 3.78 (brs, 1H) 4.27 (s, 2H), 5.02 (s, 2H), 6.19 (d, *J*=3.0 Hz, 1H), 6.33 (d, *J*=3.0 Hz, 1H), 6.64 (d, *J*=8.9 Hz, 2H), 6.79 (d, *J*=8.9 Hz, 2H).

^{1}H NMR spectrum of the final reaction mixture for **Table 3 Entry 6**:

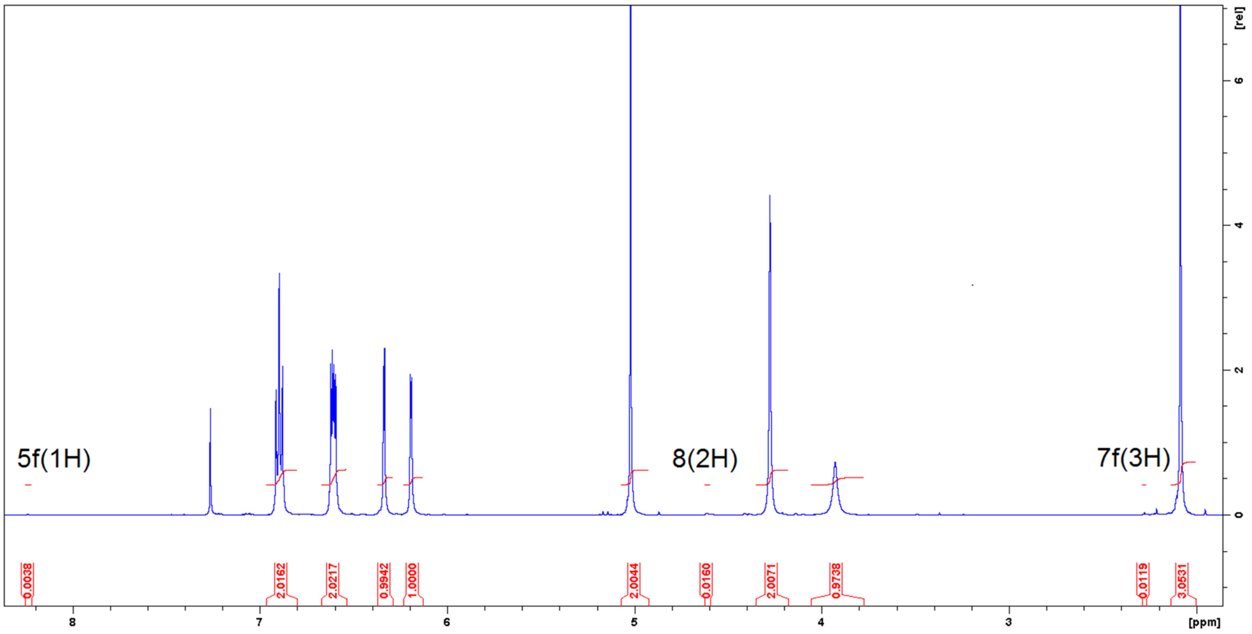

The spectrum contains peaks of compounds: **6f** (main product), **5f, 7f** and **8**.

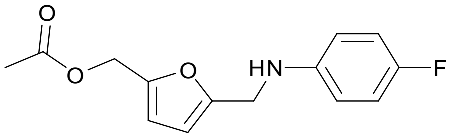

(6f)

^{1}H NMR (500 MHz, $CDCl_3$) δ ppm: 2.08 (s, 3H), 3.93 (brs, 1H) 4.27 (s, 2H), 5.02 (s, 2H), 6.19 (d, *J*=3.0 Hz, 1H), 6.33 (d, *J*=3.0 Hz, 1H), 6.57-6.64 (m, 2H), 6.85-6.94 (m, 2H).

^{1}H NMR spectrum of the final reaction mixture for **Table 3 Entry 7**:

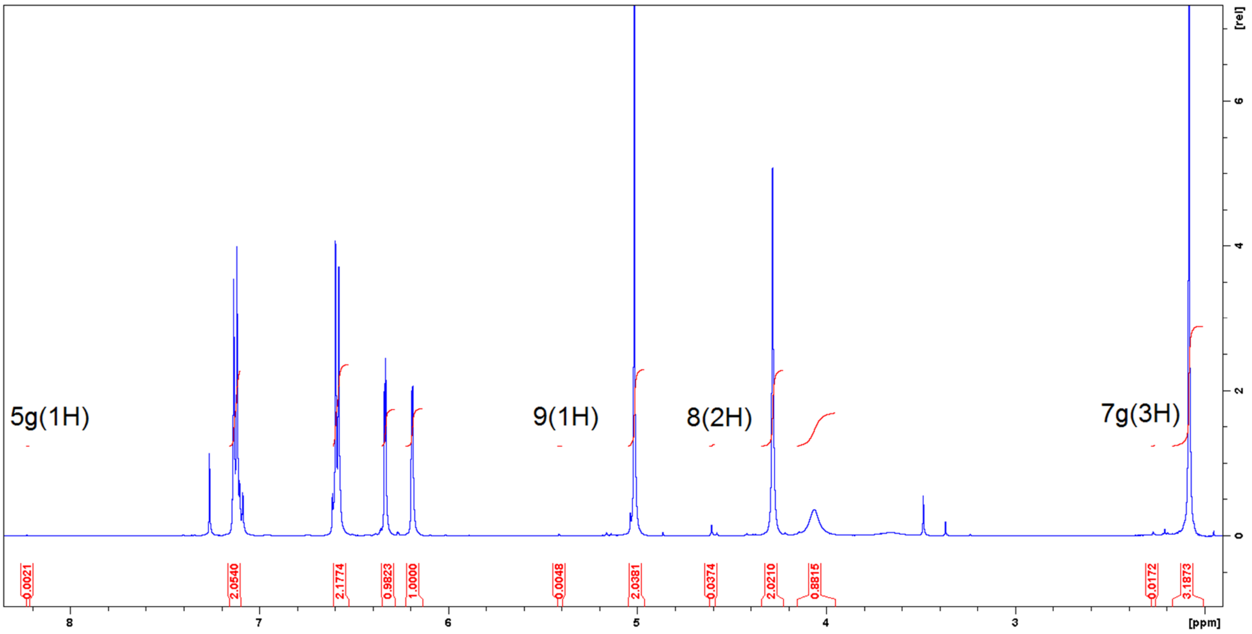

The spectrum contains peaks of compounds: **6g** (main product), **5g**, **7g**, **8** and **9**.

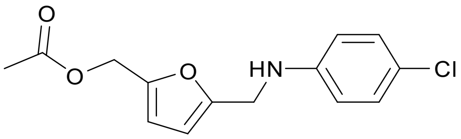

(6g)

^{1}H NMR (500 MHz, $CDCl_3$) δ ppm: 2.08 (s, 3H), 4.06 (brs, 1H), 4.28 (s, 2H), 5.01 (s, 2H), 6.19 (d, *J*=3.0 Hz, 1H), 6.33 (d, *J*=3.0 Hz, 1H), 6.59 (d, *J*=8.8 Hz, 2H), 7.12 (d, *J*=8.8 Hz, 2H).

^{1}H NMR spectrum of the final reaction mixture for **Table 3 Entry 8**:

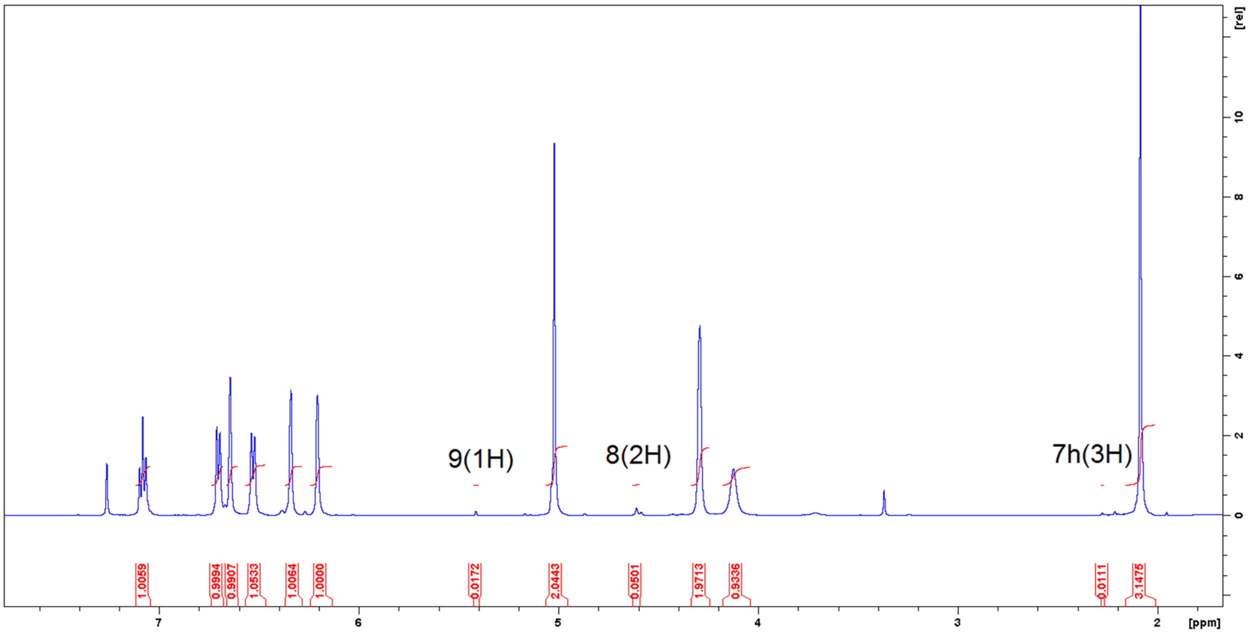

The spectrum contains peaks of compounds: **6h** (main product), **7h**, **8** and **9**.

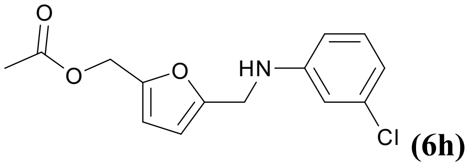

(6h)

^{1}H NMR (500 MHz, $CDCl_3$) δ ppm: 2.09 (s, 3H), 4.12 (brs, 1H), 4.29 (d, *J*=4.8 Hz, 2H), 5.02 (s, 2H), 6.21 (d, *J*=3.0 Hz, 1H), 6.34 (d, *J*=3.0 Hz, 1H), 6.49-6.55 (m, 1H), 6.64 (t, *J*=2.0 Hz, 1H), 6.68-6.72 (m, 1H), 7.08 (t, *J*=8.0 Hz, 1H).

^{1}H NMR spectrum of the final reaction mixture for **Table 3 Entry 9**:

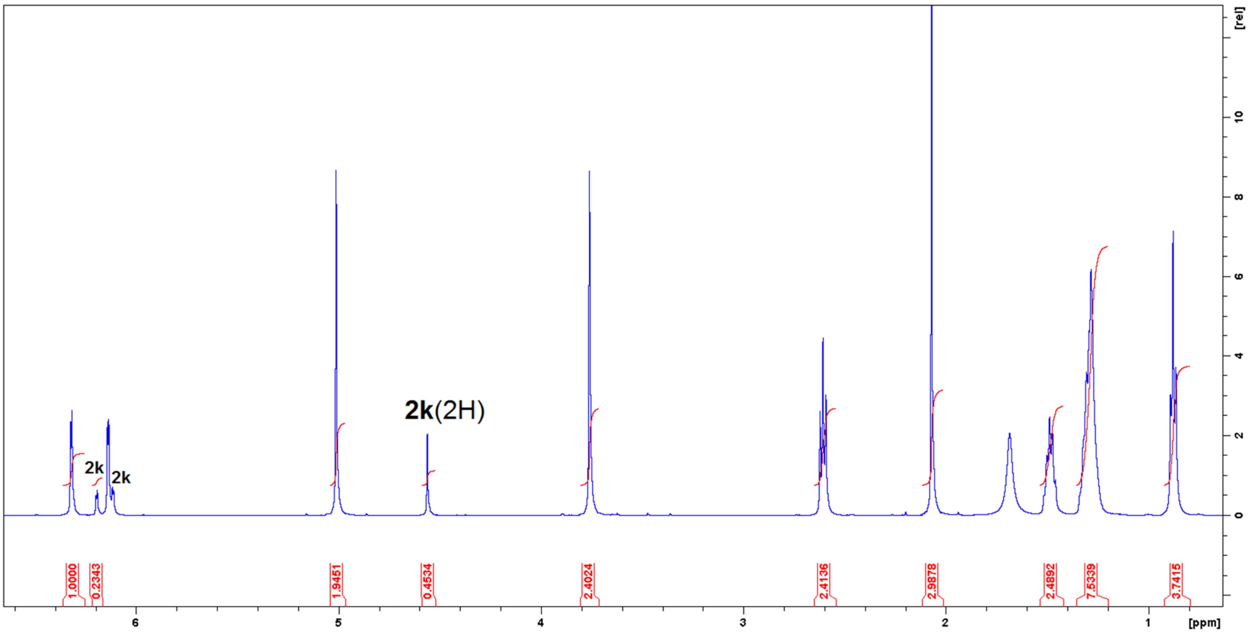

The spectrum contains peaks of compounds: **6i** (main product) and **2k**.

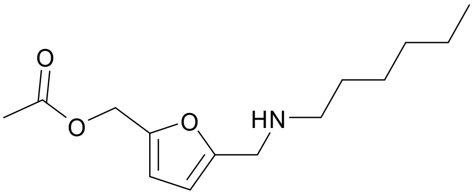

(6i)

^{1}H NMR (500 MHz, $CDCl_3$) δ ppm: 0.84-0.91 (m, 3H), 1.21-1.35 (m, 6H), 1.42-1.53 (m, 2H), 2.07 (s, 3H), 2.61 (t, *J*=7.2 Hz, 2H), 3.76 (s, 2H), 5.01 (s, 2H), 6.14 (d, *J*=3.0 Hz, 1H), 6.32 (d, *J*=3.0 Hz, 1H).
